# Identifying the Naphthalene-Based Compound 3,5-Dihydroxy 2-Napthoic Acid as a Novel Lead Compound for Designing Lactate Dehydrogenase-Specific Antibabesial Drug

**DOI:** 10.3389/fphar.2019.01663

**Published:** 2020-01-30

**Authors:** Long Yu, Xueyan Zhan, Qin Liu, Yali Sun, Muxiao Li, Yangnan Zhao, Xiaomeng An, Yu Tian, Lan He, Junlong Zhao

**Affiliations:** ^1^ State Key Laboratory of Agricultural Microbiology, College of Veterinary Medicine, Huazhong Agricultural University, Wuhan, China; ^2^ Key Laboratory of Preventive Veterinary Medicine in Hubei Province, Huazhong Agricultural University, Wuhan, China; ^3^ Key Laboratory of Animal Epidemical Disease and Infectious Zoonoses, Ministry of Agriculture, Huazhong Agricultural University, Wuhan, China

**Keywords:** *Babesia microti*, lactate dehydrogenase, naphthalene-based compound, human babesiosis, growth inhibition

## Abstract

Human babesiosis is caused by apicomplexan *Babesia* parasites, including *Babesia microti*, *Babesia crassa*, *Babesia* sp. MOI, *Babesia divergens*, *Babesia duncani*, and *Babesia venatorum*. Among them, *B. microti* is the most common cause of human and rodent babesiosis. Currently, no vaccine is available, and drugs for the treatment have high failure rates and side effects. Due to lack of a traditional tricarboxylic acid cycle (TCA cycle) and its dominant dependence on anaerobic metabolism to produce ATP, *B. microti* lactate dehydrogenase (BmLDH) was assumed to play a critical role in *B. microti* ATP supply. Our previous study demonstrated that BmLDH is a potential drug target and Arg99 is a crucial site. Herein, a molecular docking was performed based on the crystal structure of BmLDH from a series of gossypol derivatives or structural analogs to find the potent inhibitors interacting with the residue Arg99, and three naphthalene-based compounds 2,6-naphthalenedicarboxylic acid (NDCA), 1,6-dibromo-2-hydroxynapthalene 3-carboxylic acid (DBHCA), and 3,5-dihydroxy 2-napthoic acid (DHNA) were selected for further tests. Enzyme activity inhibitory experiments show that DBHCA and DHNA inhibit recombinant BmLDH (rBmLDH) catalysis with ~109-fold and ~5,000-fold selectivity over human LDH, respectively. Surface plasmon resonance (SPR) assays demonstrate that DHNA has a lower *K*_D_ value to BmLDH (3.766 x 10^−5^ M), in contrast to a higher value for DBHCA (3.988 x 10^−8^ M). A comparison of the kinetic parameters [association constant (*k*_a_) and dissociation constant (*k*_d_) values] reveals that DBHCA can bind the target faster than DHNA, while the complex of DHNA with the target dissociates slower than that of DBHCA. Both DBHCA and DHNA can inhibit the growth of *B. microti in vitro* with half-maximal inhibitory concentration (IC_50_) values of 84.83 and 85.65 μM, respectively. Cytotoxicity tests *in vitro* further indicate that DBHCA and DHNA have selectivity indexes (SI) of 2.6 and 22.1 between *B. microti* and Vero cells, respectively. Although the two naphthalene-based compounds only display modest inhibitory activity against both rBmLDH and the growth of *B. microti*, the compound DHNA features high selectivity and could serve as a novel lead compound for designing LDH-specific antibabesial drug.

## Introduction

*Babesia* species are tick-borne intraerythrocytic parasites and could cause babesiosis in humans and many animals globally (Bock et al., 2004; Westblade et al., 2017). *Babesia microti*, the smallest apicomplexan, is transmitted by ticks in both rodents and humans; however, human infection with the parasite could also be induced by blood transfusion, placenta, and solid organ transplantation (Cornillot et al., 2012; Villatoro and Karp, 2018). At present, *B. microti* is considered as an emerging zoonotic disease and widely distributed in United States of America, northeastern Eurasia, Japan, and so on (Tsuji et al., 2001; Zamoto et al., 2004; Hersh et al., 2012). No vaccines or miracle drugs are available to control and cure the parasitosis, and in general, the recommended therapies for *B. microti* are the combinations of antibacterial and antimalarial drugs, such as the combination of atovaquone with azithromycin for the treatment of mild infection or clindamycin and quinine for the treatment of severe infection (Centers for Disease Control, 1983; Krause et al., 2000). Due to the low efficiency of these drugs, the resulting drug-resistant parasite often causes relapsing and deterioration of *B. microti*, suggesting the urgency to discover new drug targets and produce new drugs against the disease (Wormser et al., 2010).

Most apicomplexan parasites derive energy from the Embden-Meyerhof pathway (EMP), and in the anaerobic metabolism, lactate dehydrogenase (LDH), an essential enzyme of anaerobic metabolism, plays a crucial role in catalyzing the reversible reaction between pyruvic acid and lactic acid, with reduced nicotinamide adenine dinucleotide (NADH) and nicotinamide adenine dinucleotide (NAD^+^) serving as co-factors, respectively (Kavanagh et al., 2004). Available studies demonstrated that the LDH enzymes of protozoans are an attackable target for drug development (Razakantoanina et al., 2000; Dando et al., 2001; Conners et al., 2005), and inhibiting the activity of these LDH enzymes can result in the death of the parasites, including *Plasmodium* spp., *Toxoplasma gondii*, *Babesia* spp., and *Cryptosporidium*. (Al-Anouti et al., 2004; Bork et al., 2004; Vivas et al., 2005; Zhang et al., 2015). Interestingly, the enzyme of *B. microti* (BmLDH) acquired by a lateral gene transfer event has been identified as a mammalian-like LDH enzyme, and is significantly different from all the other protozoans in the unusual event (Cornillot et al., 2012; Silva et al., 2016). Our previous study with the crystal structures of BmLDH indicated that the enzyme activity of BmLDH could be dramatically inhibited by gossypol and oxamate, and the residue Arg99 was crucial in the catalysis of BmLDH, but not in Human LDH-A (Yu et al., 2019). All the available reports indicate that the BmLDH could serve as a novel drug target for the development of new strategies to treat the human parasitosis.

Gossypol, a natural phenolic aldehyde extracted from the cotton plant, displays various biological activities, including anti-viral, anti-parasitic, and male contraceptive effects (Radloff et al., 1986; Royer et al., 1986; White et al., 1988). In mammalian LDHs, gossypol was observed to be a non-selective LDH inhibitor competitive with NADH binding, and the inhibition constant (*K*_i_) values were determined as 1.9 µM for LDH-A_4_, 1.4 µM for LDH-B_4_, and 4.2 µM for LDH-C_4_. In *Plasmodium falciparum*, the enzyme activity of *P. falciparum* lactate dehydrogenase (PfLDH) was inhibited competitively by gossypol with a *K*_i_ value of 0.7 μM and the growth of the parasite *in vitro* was also inhibited by gossypol with an half-maximal inhibitory concentration (IC_50_) value of 15.3 μM (Vivas et al., 2005). In *Babesia bovis*, gossypol inhibited BbLDH enzymatic activity to cause the death of *B. bovis* and its *K*_i_ and IC_50_ *in vitro* values were determined as 0.085 and 50 μM (Bork et al., 2004). In *B. microti*, the catalytic activity of BmLDH was inhibited by gossypol at 0.67 μM (*K*_i_), and the IC_50_ value of gossypol against *B. microti in vitro* was 7.07 μM (Yu et al., 2019). Previous studies showed that these derivatives of gossypol exhibited obviously lower cytotoxicity toward mammalian cells than the parent compound (Royer et al., 1991; Royer et al., 1995). Therefore, the naphthalene-based compounds, the core of the gossypol structure, could have the potential use for screening selective inhibitors of BmLDH.

Previous research on the derivatives or structural analogs of the phenolic aldehyde gossypol revealed several chemical compounds as selective human LDH inhibitors, such as the substituted 2,3-dihydroxy-1-naphthoic acid family with 200-fold selectivity over dihydroxynaphthoic acids with substituents at the 4- and 7-positions (Yu et al., 2001). In malaria parasites, although the gossypol was observed to non-selectively inhibit the catalytic activity of human LDHs and PfLDH with inhibitory constants in the low micromolar range, its derivative, 8-deoxyhemigossylic acid, has been developed to selectively inhibit PfLDH compared with human LDHs (Gomez et al., 1997).

Owing to the dependence of parasites on glycolysis system for energy generation, these enzymes play crucial roles as potential drug targets for development of anti-parasitic agents (Dunn et al., 1996). Thus far, many compounds have been reported to produce a significant effect on the enzymatic activity of the protozoan LDHs, including gossypol, derivatives or structural analogs of gossypol, oxamic acid, and azole-based compounds (Cameron et al., 2004; Choi et al., 2007; Rai et al., 2017). Therefore, screening and identifying new BmLDH inhibitors would facilitate the discovery of novel treatment strategies. The objective of the present study was to find the potent inhibitors interacting with the residue Arg99 *via* a molecular docking based on the crystal structure of BmLDH from a series of gossypol derivatives or structural analogs. Then, the screened inhibitors (naphthalene-based compounds) were further investigated by enzyme inhibition and growth inhibition experiments and cytotoxicity tests *in vitro*.

## Materials and Methods

### Compliance With Ethical Standards

This study was approved by the Scientific Ethic Committee of Huazhong Agricultural University (permit number: HZAUMO-2018-007). All mice were handled in accordance with the Animal Ethics Procedures and Guidelines of the People’s Republic of China.

### Molecular Docking

For the structure-based study, the complex crystal structure of BmLDH was obtained from Protein Data Bank (PDB accession no. 6K13) for CDOCKER docking. The structure model was further optimized by using the software (Discovery Studio 2018 Client), including removing water molecules, dopant atoms, and original ligands, cleaning geometry, adding hydrogen atoms, and defining the active site. In the CDOCKER protocol, the values of top hits, random conformations, and the orientations to refine were set as 10, respectively. In addition, the parameter of simulated annealing was switched to true and all the other parameters were maintained as defaults. In the docking study, a series of commercially available gossypol derivatives or structural analogs (16 species) were used for ligands, including 2,6-naphthalenedicarboxylic acid (PubChem CID: 14357), naphthalene-1,5-disulfonamide (PubChem CID: 96237), 1,6-dibromo-2-hydroxynapthalene 3-carboxylic acid (PubChem CID: 74502), 2,6-naphthalene disulphonic acid (PubChem CID: 11390), 3,7-dihydroxy-N-[(4-nitrophenyl)methyl]naphthalene-2-carboxamide (PubChem CID: 481499), 3,7-dihydroxy-6-(hydroxymethyl) naphthalene-2-carboxylic acid (PubChem CID: 123699198), 6,6-dithiodinicotinic acid (PubChem CID: 85040), 3,5-dihydroxy 2-napthoic acid (PubChem CID: 66637), 1,6-dihydroxynaphthalene (PubChem CID: 68463), methyl 1,6-dihydroxynaphthalene-2-carboxylate (PubChemCID: 131307105), 1-bromo-2-(bromomethyl) naphthalene (PubChemCID: 37828), dimethyl 2,6-naphthalenedicarboxylate (PubChem CID: 61225), naphthalene-1,4-dithiocarboxamide (PubChem CID: 73995732), 4,8-dimethoxy-1-naphthaldehyde (PubChem CID: 612187), 2-[5-(benzyloxy)-1H-indole-3-yl]-1,4-naphthoquinone (PubChem CID: 101865177), 2-amino-8-methoxy-1,2,3,4-tetrahydro-naphthalene-2-carboxylic acid (PubChem CID: 12526606). All the structure data format (SDF) files were obtained from PubChem (https://pubchem.ncbi.nlm.nih.gov). The software permission of discovery studio 2018 client (v18.1.100.18065) was provided by State Key Laboratory of Agricultural Microbiology of Huazhong Agricultural University (Wuhan, Hubei, China).

### Parasites

*B. microti* ATCC PRA-99™ strain was provided by the National Institute of Parasitic Diseases, Chinese Center for Disease Control and Prevention (Shanghai, China) and preserved in liquid nitrogen with the additive of dimethyl sulfoxide (DMSO) in the State Key Laboratory of Agricultural Microbiology, Huazhong Agricultural University, China.

### Preparation of Genomic Deoxyribonucleic Acid

Blood sample was collected from the tail of BALB/c mouse into 1.5 ml centrifuge tubes containing EDTA-K_2_ (Sigma, Shanghai, China) at day 7 post-infection. Genomic DNA (gDNA) of *B. microti* was extracted by using the QIAamp DNA Blood Mini Kit (Qiagen, Hilden, Germany) according to the manufacturer’s instruction and stored at –20°C until usage.

### Construction of Recombinant Plasmid, Protein Expression, and Purification

Primer pairs with the *BamH* I and *Xho* I restriction sites were designed based on the full-length open reading frame (ORF) of BmLDH obtained from the GenBank database under the accession number MN102392 (**Table 1**). The PCR thermo-cycling was done in a 50 µl reaction volume including 50 ng gDNA, 0.2 µM primers, 0.2 mM deoxyribonucleotide triphosphates (dNTPs), 10 µl 5 × *TransStart* ^®^ *KD* Plus Buffer, and 1 U *TransStart* ^®^ *KD* Plus DNA Polymerase (TransGen Biotech, Beijing, China). The PCR reaction was performed at 94°C for 5 min, followed by 35 cycles of 94°C for 30 s, 58°C for 30 s, 68°C for 1 min, and finally at 68°C for 10 min. The resulting PCR product was purified by using EasyPure^®^ PCR Purification Kit (TransGEN, Beijing, China), and then cloned into a pET-28a expression vector. The plasmid construct was confirmed by DNA sequencing.

**Table 1:** Oligonucleotide primers used for amplification of the open reading frame of the *Babesia microti* lactate dehydrogenase gene.

Primers	Primer sequences (5’-3’)	Restriction enzyme
BmLDH-F	ACGGATCCATGCATTCGTTAAAAGAAG	*BamH* I
BmLDH-R	GCCTCGAGTTATAGTTGGATATCTTTCTG	*Xho* I

The sequence-correct recombinant plasmid was transformed into the *Escherichia coli* BL21 expressing host cells. The transformed cells were cultured at 37°C in LB medium containing 0.1 mg/ml (1:1000 dilution) kanamycin for 3 h. When the culture density reached an optical density of 0.6 to 0.8 at 600 nm (OD_600_), induction was performed with 0.8 mM IPTG (Biosharp, Anhui, China), and the growth of cells continued for additional 12 h at 28°C before harvesting.

For protein purification, the induced cells were harvested by centrifugation at 7,000 rpm for 10 min in a high-speed refrigerated centrifuge (Hitachi, Tokyo, Japan), followed by re-suspending with His binding buffer (300 mM NaCl, 10 mM Tris-base, 50 mM NaH_2_PO_4_·2H_2_O, 10 mM imidazole, pH7.5) and lysis by passage through an high-pressure homogenizer at 1,000 bar. After centrifugation at 10,000 rpm/min for 10 min, the supernatant was filtered through a 0.45-um-pore-size filter and loaded onto Ni Sepharose™ High Performance affinity matrix (GE Healthcare, Uppsala, Sweden). The proteins were eluted gradiently with elution buffer (20–400 mM imidazole) and stored at −80°C. His tag on the N-terminus was not cleaved.

### Western Blot Analysis

According to the standard method, the purified rBmLDH and *B. microti* lysates were separately subjected to 12% sodium dodecyl sulfate polyacrylamide gel electrophoresis (SDS-PAGE), followed by electroblotting onto a nitrocellulose (NC) membrane. Next, the Western blot membranes were blocked with 0.05% Tween 20 in TBS (TBST) plus 1% BSA for 8 h at 4°C. After five washes with TBST, the NC membranes were separately probed by using *B. microti*-infected positive serum, anti-BmLDH monoclonal antibody (McAb), and specific pathogen-free (SPF) mouse serum (1:200, diluted with TBST) at 37°C for 1 h. Then, the NC membranes were washed five times with TBST, followed by incubation at 37°C for 1 h with horseradish peroxidase (HRP) labeled goat anti-mouse IgG secondary antibody diluted with TBST (1:1,000). After washing five times, the NC membranes were visualized using the ECL method.

### Enzyme Inhibition Analysis

The enzyme activity of rBmLDH was analyzed using Lactate Dehydrogenase Activity Assay Kit (Sigma-Aldrich, Shanghai, China) according to the manufacturer’s instruction. Briefly, the enzymatic activity of BmLDH was detected by catalyzing the conversion of lactate to pyruvate, with concomitant reduction of NAD^+^ to NADH. The coupled reaction was followed by measuring absorbance at 450 nm and at 37°C. Three naphthalene-based compounds [2,6-naphthalenedicarboxylic acid (NDCA), 1,6-dibromo-2-hydroxynapthalene 3-carboxylic acid (DBHCA), and 3,5-dihydroxy 2-napthoic acid (DHNA)] were purchased from Sigma-Aldrich (Shanghai, China) with 99% purity for NDCA and 97% purity for DBHCA and DHNA. In the inhibitory analysis, three naphthalene-based compounds were prepared as 100 mM stock solution in 100% dimethylsulfoxide (DMSO) (**Table 2**). The percentage inhibition of BmLDH was determined separately at various concentrations (50–500 µM). As a positive control, the phenolic aldehyde gossypol at 0.7 µM was used to confirm the validity of the experimental results (Vudriko et al., 2014). The concentrations of the two compounds that inhibited NADH production by 50% (IC_50_ values) were calculated separately using GraphPad prism5. The optimal additive amount of rBmLDH was 100 ng. The final concentrations of DMSO did not influence the BmLDH activity as determined in a preliminary experiment and all the experiments were repeated three times.

**Table 2:** *In vitro* evaluation of three naphthalene-based compounds against *Babesia microti* at the asexual blood stage, cytotoxicity in Vero cells, and selectivity index (SI).

Structure	Compound ID	BmLDH IC_50_ (mM)	PfLDH IC_50_ (mM)	HmLDH IC_50_ (mM)	PRA-99 IC_50_ (mM)	CC_50_ Vero (mM)	SI
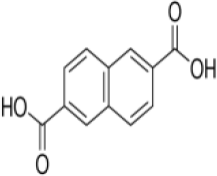	2,6-Naphthalenedicarboxylic acid (NDCA)	> 0.5	5.1*	1.4*	−	−	−
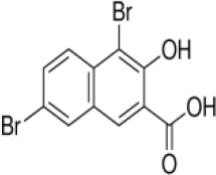	1,6-Dibromo-2-hydroxynapthalene 3-carboxylic acid (DBHCA)	0.054	0.31*	5.9*	0.085	0.217	2.6
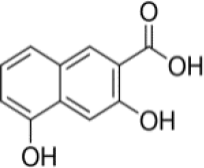	3,5-Dihydroxy 2-napthoic acid (DHNA)	0.030	1.7*	150*	0.086	1.9	22.1

*These values were acquired from R. Conners et al., 2005. BmLDH, Babesia microti lactate dehydrogenase; PfLDH, Plasmodium falciparum lactate dehydrogenase; HmLDH, human lactate dehydrogenase; PRA-99, B. microti ATCC PRA-99™ strain; IC_50_, half-maximal inhibitory concentration; CC_50_, half-maximal cytotoxic concentration.

### Surface Plasmon Resonance Assays

The binding properties of the rBmLDH against DBHCA and DHNA were measured separately by SPR experiment with a Biacore T200 system (GE Healthcare, Uppsala, Sweden). Briefly, a sensor chip CM5 was put into a Biacore T200 system and washed with the running buffer (PBST+0.5% DMSO, pH7.5). The CM5 chip was activated by injecting the mixture of 0.1 M *N*-hydroxysuccinimide (NHS) and 0.4 M 1-ethyl-3-(3-dimethylaminopropyl) carbodiimide hydrochloride (EDC) (1:1, v/v) at a flow rate of 10 μl·min^−1^ for 20 min. Then, the rBmLDH diluted to 10 μg·ml^−1^ with 10 mM sodium acetate buffer (pH 4.0, 4.5, 5.0, and 5.5) was covalently immobilized on the CM5 chip at a flow rate of 10 μl·min^−1^ until the immobilization level of BmLDH reached 2,100 resonance units (RU). Ethanolamine solution was injected to block the remaining unreacted esters. Flow cell 1 was activated and blocked as the reference surface, while flow cell 2 served as the experimental surface. The 100 mM stocks of the two small molecule compounds were further diluted with PBST into 500, 250, 125, 62.5, 31.25, and 15.625 μM. The surface of CM5 sensor chip was fully regenerated using 10 mM glycine pH2.5 at a flow rate of 30 μl min^−1^ for 30 s. The SPR kinetic experiments were performed three times and the values were presented as the mean ± SD of three independent experiments.

### Inhibitory Effect of Two Naphthalene-Based Compounds Against the Growth of *Babesia Microti* *In Vitro*

Three BALB/c mice were intraperitoneally injected with 1 x 10^7^ parasites (100 μl fresh blood), and their blood was collected from the tail when parasitemia reached ~60% at day 7 post-infection. The growth inhibition assay of *B. microti in vitro* was performed by incubating freshly isolated infected mouse red blood cells (RBCs) with varying concentrations of the drugs. Briefly, 25 μl infected mouse RBCs was diluted with 5 μl non-infected mouse RBCs and 10 μl non-infected human RBCs to achieve ~3% parasitemia in a total of 40 μl and suspended in 110 μl of a growth medium (20% bovine serum and 80% medium HL20) containing the indicated concentrations of drugs (50, 100, and 250 μM). The cultures were maintained at 37°C for 72 h in a gas mixture of 2% O_2_, 5% CO_2_, and 93% N_2_. Two naphthalene-based compounds DBHCA and DHNA were prepared as 100 mM stock solutions in DMSO and further diluted with growth medium. Each concentration of the compounds was tested in triplicate, while three wells received a drug-free culture medium as a control. 10 μM diminazene aceturate (DA) was selected as a positive control. Parasitemia was determined at 72 h post-treatment by enumerating a total of at least 1,000 RBCs in a Giemsa-stained thin smear. Differences in the percent parasitemia were statistically analyzed using one-way ANOVA analysis, and the IC_50_ values were calculated manually for obtaining more reliable data (Käber method). The hemolysis of DBHCA and DHNA against *B. microti*-free erythrocytes was monitored by cell counting chamber at 72 h post-treatment. All the experiments were separately repeated three times.

### Cytotoxicity Test by the 3-(4,5-Dimethythiazol-2-yi)-2,5-Diphenyl Tetrazolium Bromide Method

Mycoplasma-free Vero cell line (ATCC-CCL-81, immortalized cells) was preserved in liquid nitrogen with the additive of DMSO in the State Key Laboratory of Agricultural Microbiology, Huazhong Agricultural University, China. The cytotoxicities of DBHCA and DHNA were determined in the kidney cells of the African green monkey (Vero cells) using the 3-(4,5-dimethythiazol-2-yl)-2,5-diphenyl tetrazolium bromide (MTT) method according to a previous report (Pobsuk et al., 2019). Briefly, Vero cells (100 µl, 5,000 cells/well) were cultured in Dulbecco’s modified Eagle medium (DMEM) medium containing 10% fetal bovine serum (FBS) and 100 U/ml Penicillin-Streptomycin at 37°C with 5% CO_2_ for 24 h and then with different concentrations of DBHCA or DHNA for 72 h. After adding 10 µl MTT (5 mg/ml in medium) into each test well, the plate was further incubated for another 2 h, followed by removing the culture from the incubator and discarding the medium. The resulting formazan crystals were dissolved by adding 100 µl of MTT solubilization solution (10% Triton X-100 plus and 0.1 N HCl in anhydrous isopropanol). The plate was gently mixed on a gyratory shaker for 10 min and the absorbances were measured at a wavelength of 570 nm using a microplate reader (BioTek, Vermont, USA). The background absorbances of the multi-well plate at 690 nm were subtracted from the 570 nm measurement. The DBHCA and DHNA compounds were prepared as 100 mM and 1 M of the stock solutions in 100% DMSO. In these tests, 3 µM of doxorubicin and 0.5% DMSO were used as the positive control and negative control, respectively. The CC_50_ values were calculated using GraphPad Prism 5 (San Diego, CA, USA), and all the cytotoxicity tests were performed three times.

## Results

### Molecular Models of the Compounds 2,6-Naphthalenedicarboxylic Acid, 1,6-Dibromo-2-Hydroxynapthalene 3-Carboxylic Acid, and 3,5-Dihydroxy 2-Napthoic Acid With *Babesia microti* Lactate Dehydrogenase

**Figure 1:**
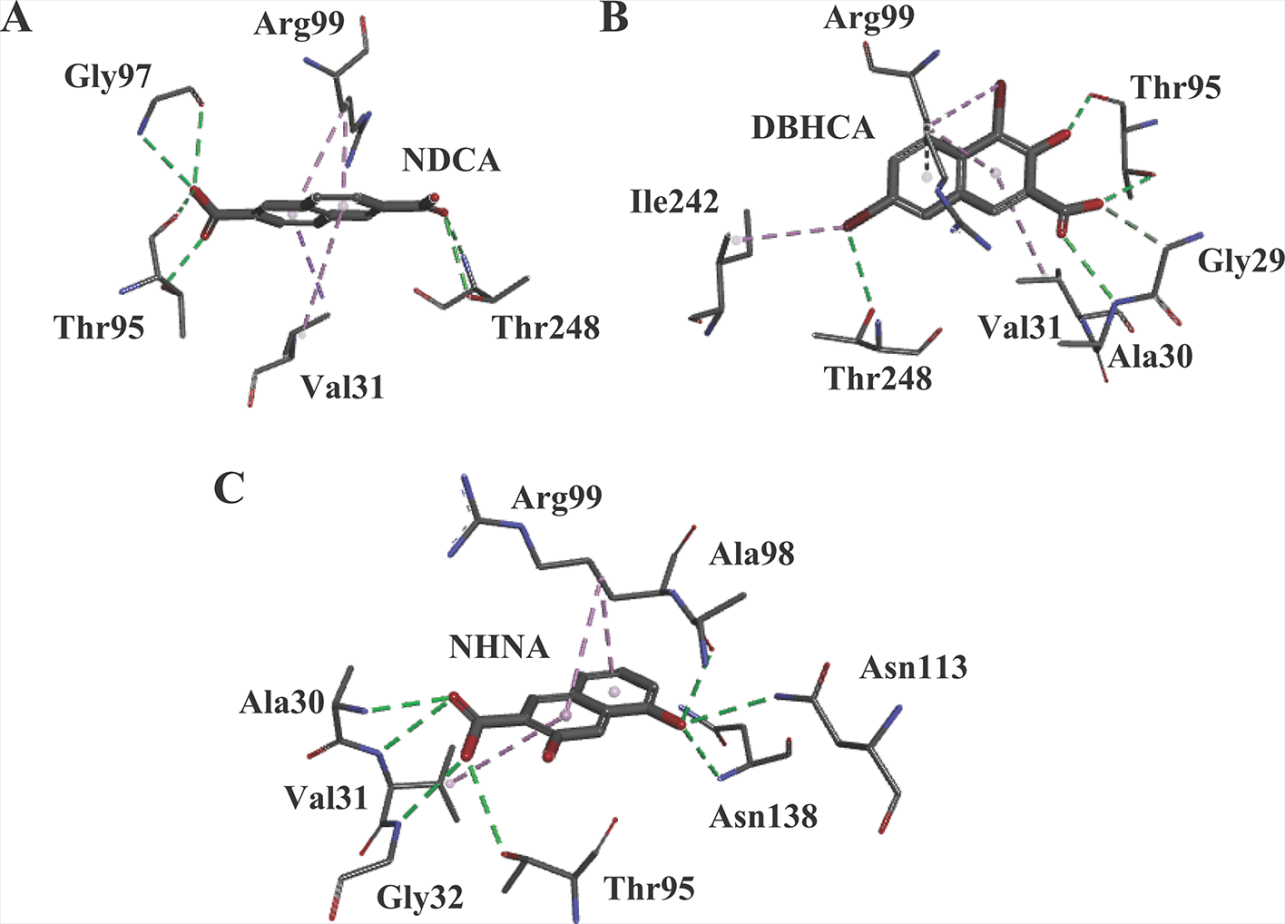
The results of molecular docking. **(A)** Molecular model of the compound 2,6-naphthalenedicarboxylic acid (NDCA) with *Babesia microti* lactate dehydrogenase (BmLDH). The (panel **A**) displays the distribution of hydrogen bonds and hydrophobic interactions at the NDCA interface. **(B)** Molecular model of the compound 1,6-dibromo-2-hydroxynapthalene 3-carboxylic acid (DBHCA) with BmLDH. The (panel **B**) displays distribution of hydrogen bonds and hydrophobic interactions at the DBHCA interface. **(C)** Molecular model of the compound 3,5-dihydroxy 2-napthoic acid (DHNA) with BmLDH. The (panel **C**) displays the distribution of hydrogen bonds and hydrophobic interactions at the DHNA interface. All interacting amino acids in the binding networks are shown as stick diagrams and labeled. The conventional hydrogen bonds (green) and Pi-alkyl bonds (violet) are shown as dashed lines between the respective donor and acceptor atoms.

Our previous study based on the crystal structures of BmLDH indicated that the mutation of residue Arg99 to Ala significantly reduced the enzyme activity of BmLDH (up to 86%), but had no impact on Human LDH-A (Yu et al., 2019). For testing the probability of BmLDH as a selective drug target, the potent inhibitors interacting with the residue Arg99 were screened *via* a molecular docking based on the crystal structure of BmLDH from a series of gossypol derivatives or structural analogs. In CDOCKER analysis, these compounds were located by DS software for simulating the binding of ligands, and three ligands NDCA, DBHCA, and DHNA interacting with the Arg99 of BmLDH while inhibiting human LDH with a high IC_50_ value were selected for subsequent tests (**Figures 1A–C**).

### Cloning *Babesia microti* Lactate Dehydrogenase Gene and Expression of the Recombinant *Babesia microti* Lactate Dehydrogenase

**Figure 2:**
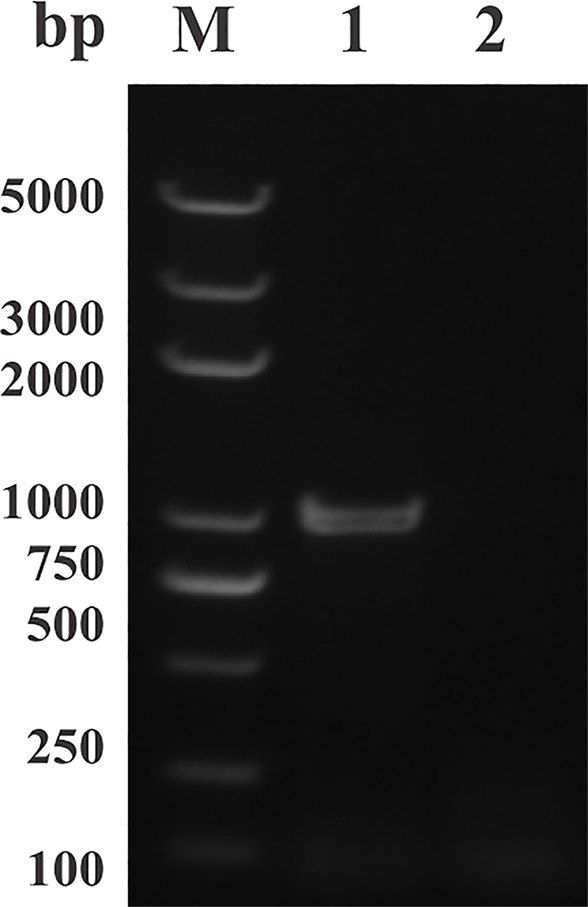
PCR results of amplifying the open reading frame (ORF) of *Babesia microti* lactate dehydrogenase (BmLDH) from *B. microti* genomic DNA (gDNA). Lane M, molecular weight marker; lane 1, the full length ORF of BmLDH (999 bp); lane 2, negative control.

**Figure 3:**
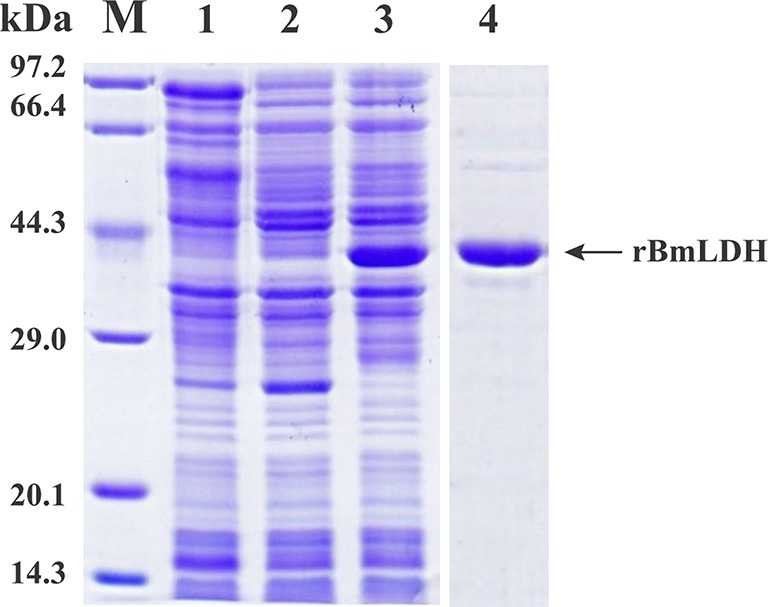
Sodium dodecyl sulfate polyacrylamide gel electrophoresis (SDS-PAGE) of prokaryotic expression stained by Coomassie blue. Induced pET-28a empty vector plasmid (lane 1), non-induced control (lane 2), induced protein (lane 3), and purified *Babesia microti* lactate dehydrogenase (BmLDH) (lane 4) were indicated by SDS-PAGE analysis.

The open reading frame (ORF) sequence of BmLDH obtained from *B. microti* gDNA by PCR had a full length 999 bp (**Figure 2**). The intact ORF encoding BmLDH was expressed in *E. coli* BL21 (DE3) and the size of recombinant BmLDH (rBmLDH) was ~37 kDa (**Figure 3**, lane 3). The rBmLDH with the N-terminal His-tag was purified by Ni sepharose for subsequent enzyme activity inhibitory analysis (**Figure 3**, lane 4).

### Recombinant *Babesia microti* Lactate Dehydrogenase Against *Babesia microti* Infected Serum and Mouse Negative Serum

**Figure 4:**
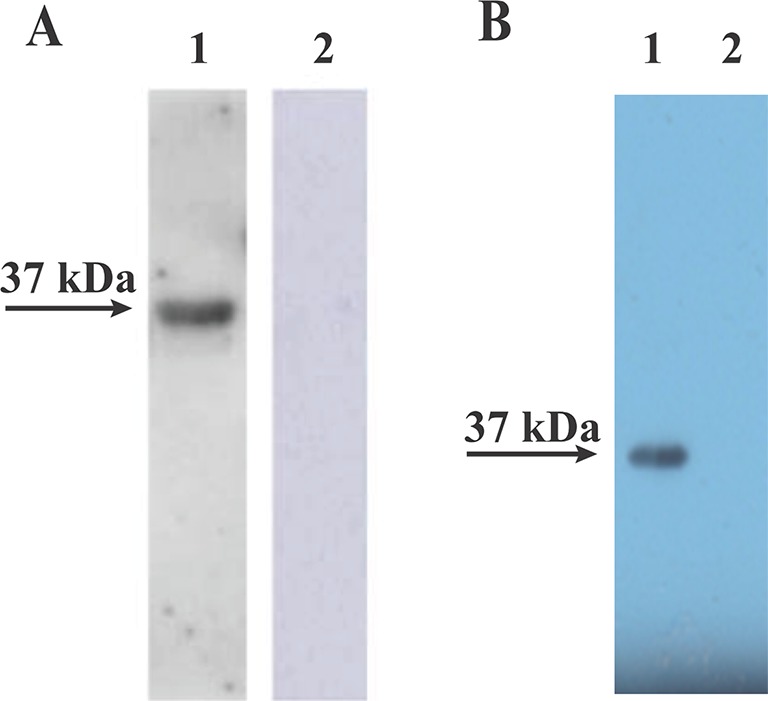
Western blot analysis. **(A)** The recombinant (*Babesia microti* lactate dehydrogenase) rBmLDH expressed in *Escherichia coli* BL21 was separately reacted with *B. microti*-infected mouse serum (lane 1) and SPF mouse serum (lane 2). **(B)** The monoclonal antibody (McAb) of BmLDH reacted with the lysates of *B. microti*-infected red blood cells (RBCs) (lane 1) and non-infected RBCs (lane 2).

To verify that the rBmLDH expressed in *E. coli* BL21 was precisely encoded by the ORF of BmLDH, an immunoblotting analysis was performed in this study. The Western blot analysis result indicated that the rBmLDH differentiated between *B. microti* positive and negative sera from BALB/c mouse. A ~37 kDa band agreeing with the rBmLDH size was revealed by using *B. microti* infected mouse positive serum, and no signal appeared with SPF serum as negative control (**Figure 4A**). Furthermore, the monocloncal antibody against BmLDH recognized the native BmLDH (~37 kDa) in extracts from *B. microti*-infected mouse RBCs, but not in the control (uninfected mouse erythrocytes) (**Figure 4B**).

### Inhibition of *Babesia microti* Lactate Dehydrogenase Activity

**Figure 5:**
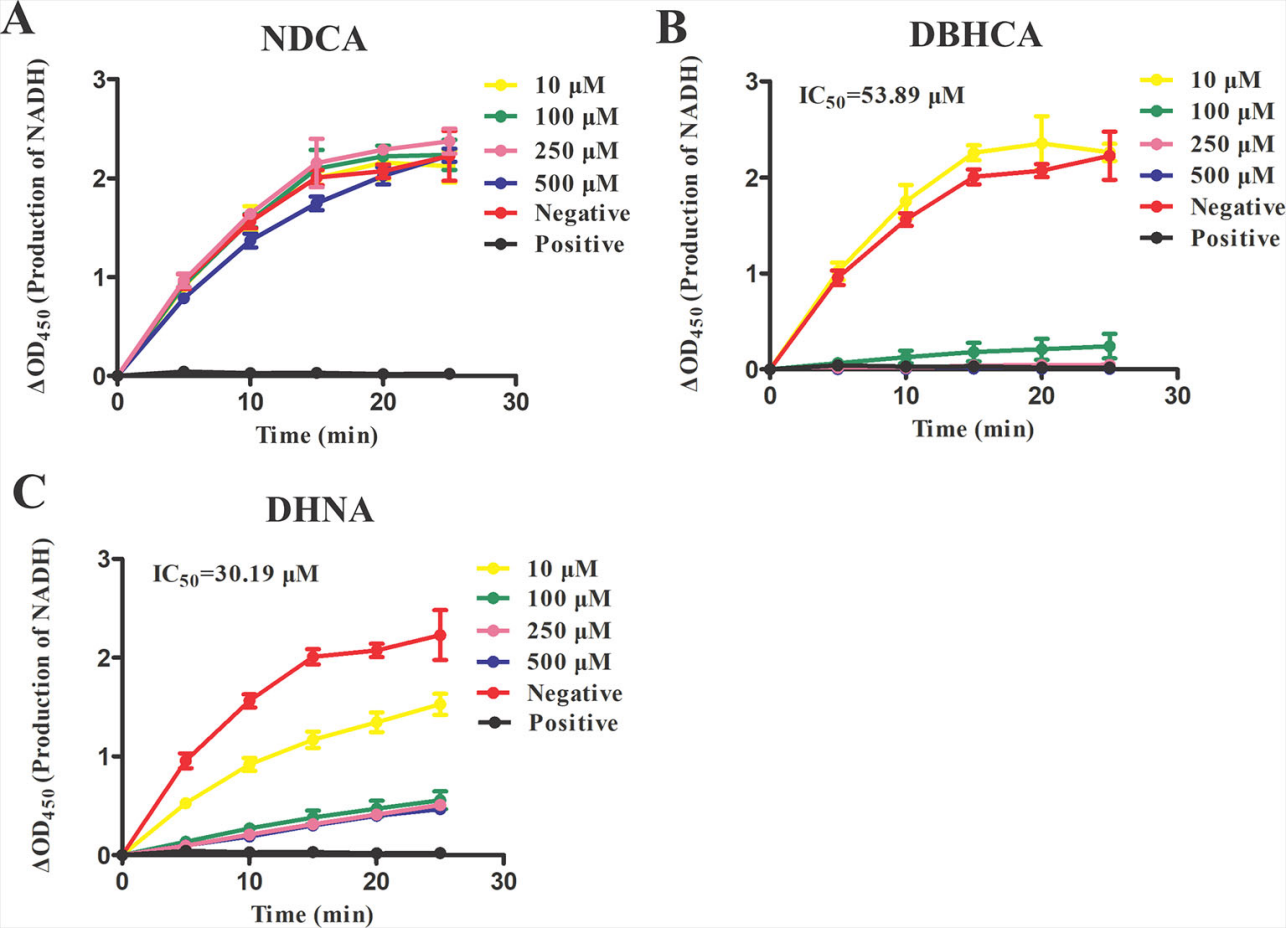
Inhibitory analysis of *Babesia microti* lactate dehydrogenase (BmLDH) activity. The half-maximal inhibitory concentration (IC_50_) values were determined in the presence of various concentrations of the three naphthalene-based compounds. The inhibition of catalytic reactions was evaluated by adding 0, 10, 100, 250 and 500 μM of 2,6-naphthalenedicarboxylic acid (NDCA) **(A)**, 1,6-dibromo-2-hydroxynapthalene 3-carboxylic acid (DBHCA) **(B)**, and 3,5-dihydroxy 2-napthoic acid (DHNA) **(C)** separately into 100 μl reaction systems. The absorbance T_initia_ and T_final_ (5 min) was measured at 450 nm. XY graphs were drawn by linear regression and error bars represent means ± SD of three independent experiments.

For finding novel BmLDH inhibitors, three naphthalene-based compounds (NDCA DBHCA, and DHNA) were used to explore their inhibitory effects against BmLDH. The results revealed that the rBmLDH catalyzed the conversion of lactate and pyruvate, and the catalysis was moderately inhibited by the two naphthalene-based compounds at micromolar concentrations. The positive control 0.7 μM gossypol ~100% inhibited the catalytic activity of BmLDH (**Figures 5A–C**). The IC_50_ values of DBHCA and DHNA were determined as 53.89 ± 13.28 and 30.19 ± 8.49 μM, respectively (**Figures 5B, C**). However, the NDCA showed no inhibitory effect on the BmLDH activity, even at the high concentration of 500 μM (**Figure 5A**).

### Binding Kinetics of *Babesia microti* Lactate Dehydrogenase

**Figure 6:**
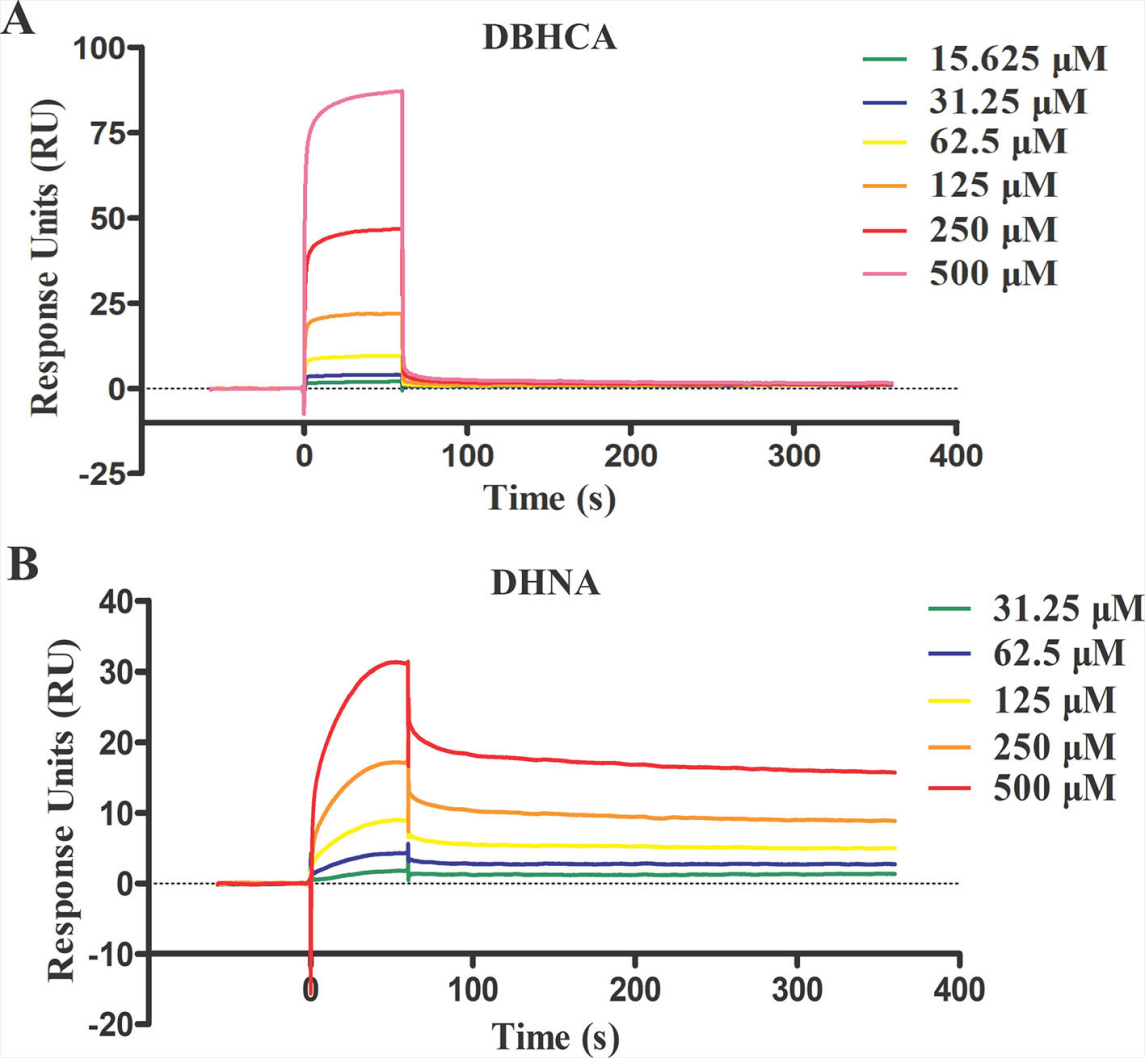
Surface plasmon resonance (SPR) kinetic sensorgrams. Two naphthoic acid compounds 1,6-dibromo-2-hydroxynapthalene 3-carboxylic acid (DBHCA) **(A)** and 3,5-dihydroxy 2-napthoic acid (DHNA) **(B)** bind to *Babesia microti* lactate dehydrogenase (BmLDH) at different concentrations (500, 250, 125, 62.5, 31.25, and 15.625 μM), respectively. The responses were reference subtracted and blank deducted.

For binding kinetic assays, standard solutions of two naphthalene-based compounds (DBHCA and DHNA) at different concentrations were tested and the data were fitted with a 1:1 binding kinetic model. The SPR kinetic sensorgrams were presented in **Figure 6** and the binding kinetic values of DBHCA and DHNA against BmLDH were shown in **Table 3**. Comparatively, DHNA showed a lower *K*_D_ value to BmLDH (3.766 x 10^−5^ M), but DBHCA exhibited a higher value with BmLDH (3.988 x 10^−8^ M). A comparison of kinetic parameters (*k*_a_ and *k*_d_) indicated that DBHCA bound the target faster than the DHNA, while the complex of DHNA with the target dissociated slower than that of DBHCA.

**Table 3:** Binding kinetic characterization of the two naphthoic acids.

Ligand	Compounds	*k*_a_ (1/Ms)	*k*_d_ (1/s)	*K*_D_ (M)
BmLDH	1,6-Dibromo-2-hydroxynapthalene 3-carboxylic acid (DBHCA)	3.766 × 10^4^ ± 8.57 × 10^2^	1.502 × 10^−3^ ± 1.76 × 10^−5^	3.988 × 10^−8^ ± 1.42 × 10^−9^
BmLDH	3,5-Dihydroxy 2-napthoic acid (DHNA)	20.49 ± 4.18 × 10^−1^	7.718 × 10^−4^ ± 6.55 × 10^−5^	3.766 × 10^−5^ ± 1.96 × 10^−6^

### Two Lactate Dehydrogenase Inhibitors Interfere With the Growth of *Babesia microti* *In Vitro*

**Figure 7:**
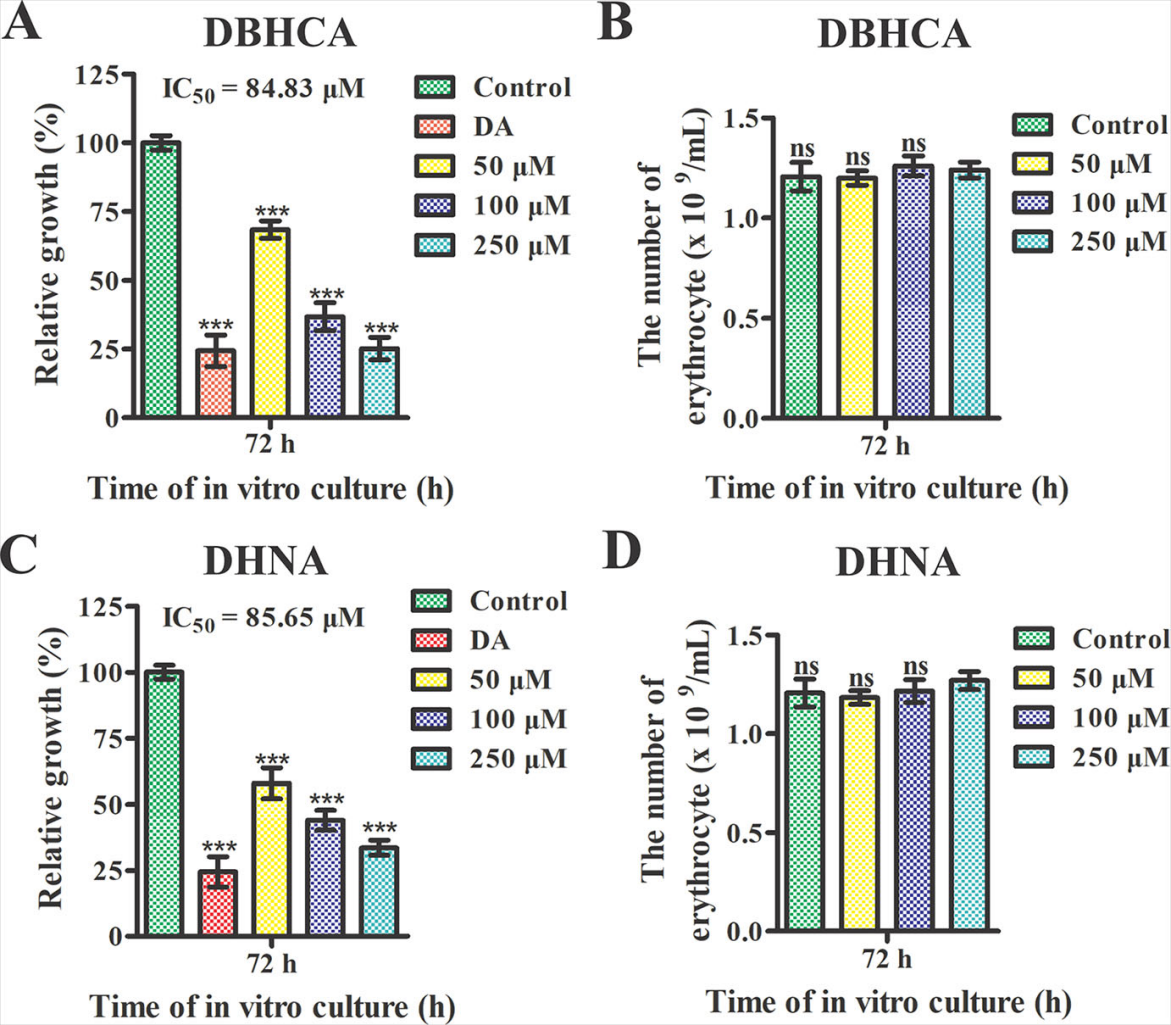
Growth inhibition analysis of *Babesia microti* by 1,6-dibromo-2-hydroxynapthalene 3-carboxylic acid (DBHCA) and 3,5-dihydroxy 2-napthoic acid (DHNA). **(A, C)** The 1,6-dibromo-2-hydroxynapthalene 3-carboxylic acid (DBHCA) and DHNA against *B. microti*-infected erythrocytes at 72 h post-treatment. The growth rates of *B. microti in vitro* were determined in the presence of different concentrations of DBHCA and DHNA. Ten micrograms of diminazene aceturate (DA) was used as the positive control. **(B, D)** Hemolytic activities of the DBHCA and DHNA against *B. microti*-noninfected erythrocytes at 72 h post-treatment. The hemolyses were monitored in the presence of different concentrations of DBHCA and DHNA. Asterisks indicate a significant difference between the drug groups and the control (three asterisks represent P < 0.001). Error bars represent means ± SD (n = 3 independent experiments), and all charts were produced using GraphPad Prism 5. Ns, not significant.

We further tested the effect of DBHCA and DHNA on the *in vitro* cultivation of *B. microti*. Parasitemia was counted at 72 h post-treatment by microscopy. The positive control, 10 μM DA inhibited the growth of the parasite by ~75.54%. Compared to the control group, the two inhibitors exhibited anti-*B. microti* activity at a low micromolar concentration (**Figures 7A, C**). The IC_50_ values of DBHCA and DHNA were calculated as 84.83 ± 6.96 and 85.65 ± 7.23 μM, respectively. Both DBHCA and DHNA showed no cellular hemolysis at drug levels up to 250 µM (**Figures 7B, D**).

### Mammalian Cytotoxicity of the Two Naphthalene-Based Compounds

**Figure 8:**
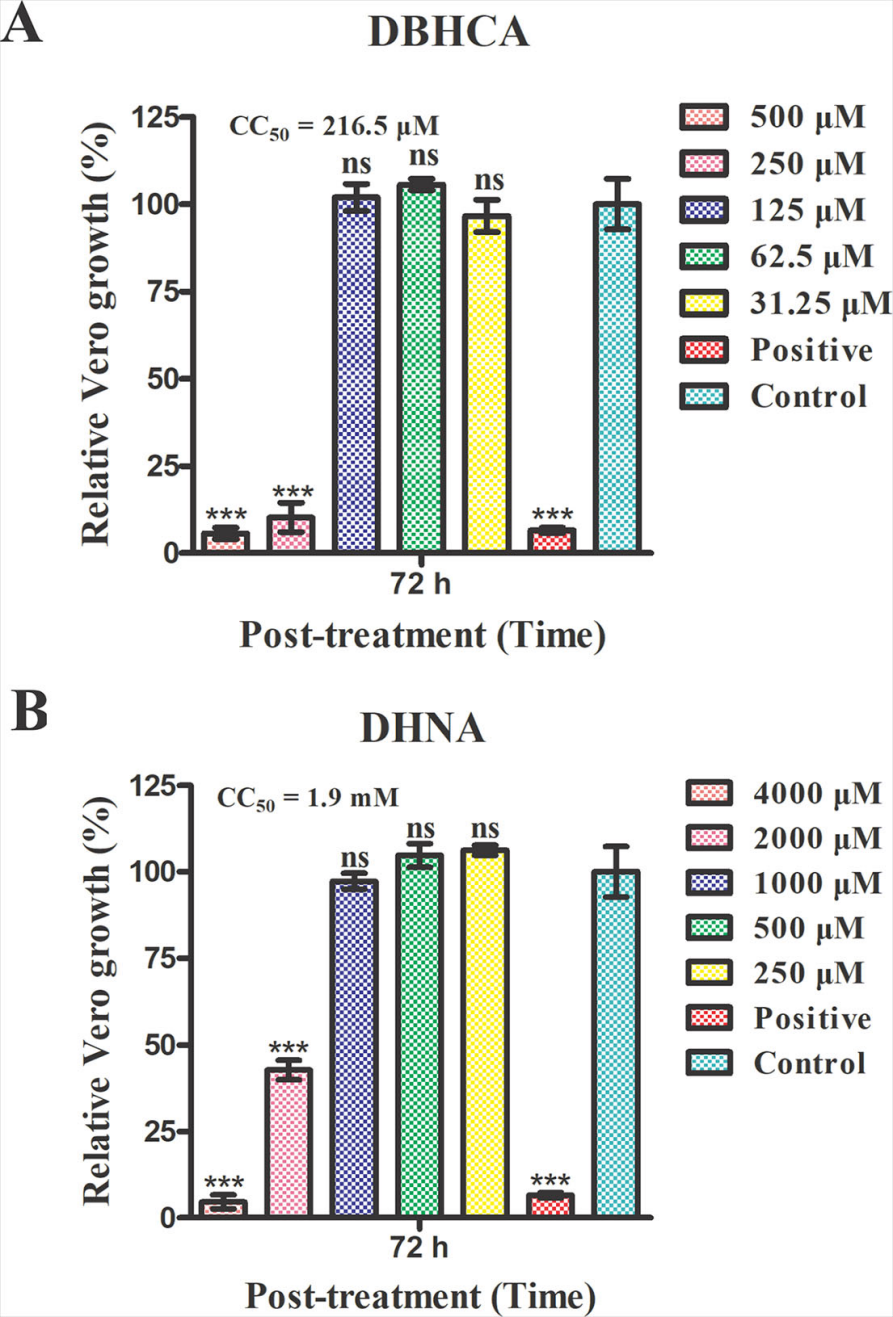
Mammalian cytotoxicities of the compounds 1,6-dibromo-2-hydroxynapthalene 3-carboxylic acid (DBHCA) and 3,5-dihydroxy 2-napthoic acid (DHNA) against Vero cell line. **(A)** The different concentrations (500, 250, 125, 61.5, and 31.25 μM) of DBHCA against Vero cells *in vitro*. The viabilities of Vero cells were monitored using 3-(4,5-dimethythiazol-2-yl)-2,5-diphenyl tetrazolium bromide (MTT) method at 72 h post-treatment. **(B)** The different concentrations (4000, 2000, 1000, 500 and 250 μM) of DHNA against Vero cells *in vitro*. The cytotoxic effects on Vero cells were observed by MTT assay at 72 h post-treatment. Differences in the cell relative growth rate were statistically analyzed using one-way ANOVA analysis and the asterisks indicate a significant difference between the drug groups and the negative control (three asterisks represent P < 0.001). Error bars represent means ± SD (n = 3 independent experiments), and all charts were produced using GraphPad Prism 5. Ns, not significant.

The above results showed that the compounds DBHCA and DHNA could inhibit both rBmLDH catalysis and the growth of *B. microti in vitro*, but their cytotoxic risk has not yet been evaluated. In this study, the cytotoxicity of DBHCA and DHNA was assessed in Vero cells using the MTT based method. The cytotoxicity test results indicated that the compound DBHCA with a selectivity indexes (SI) of 2.6 had a stronger cytotoxic activity against Vero cells, and its half-maximal cytotoxic concentration (CC_50_) value (50% cytotoxicity concentration) was 216.5 ± 18.03 μM (**Figure 8A**), while the compound DHNA with a SI of 22.1 had a lower cytotoxic effect on Vero cells, and its CC_50_ value was calculated as 1.9 ± 0.1 mM (**Figure 8B**). The positive control doxorubicin inhibited the growth of Vero cells by ~95% at 3 µM while the 0.5% DMSO as the negative control revealed no cytotoxicity against Vero cells.

## Discussion

Previous studies have shown that gossypol and its derivatives are non-selective drugs and interact with a great range of oxidoreductases, imposing restrictions on the usage of these drugs as effective medicaments for treatment of different parasitizations by apicomplexan (Montamat et al., 1982; Deck et al., 1998; Razakantoanina et al., 2000). Gossypol and its derivatives and analogs have been reported to compete with co-factors in the nicotinamide binding site of PfLDH and display powerful effects on the enzyme, but the core of the gossypol structure exhibits weak inhibition (Conners et al., 2005). Herein, three naphthalene-based compounds (NDCA, DBHCA, and DHNA), the core of the gossypol structure, were used for exploring new BmLDH inhibitors and therapeutic drugs for babesiosis. Interestingly, rBmLDH activity was 100% inhibited by DHNA at ~60 μM, and the DHNA displayed ~5,000-fold selectivity over human LDH and ~57-fold selectivity over PfLDH (**Table 2**)_._ Cytotoxicity tests demonstrated that the compounds DBHCA and DHNA had SI of 2.6 and 22.1 between *B. microti* and Vero cells. The results suggest that the DHNA could be a better candidate of lead compound for developing new anti-*Babesia* drug with high affinity and selectivity than that of the DBHCA.

In this study, we characterized the binding properties of DBHCA and DHNA against BmLDH by SPR experiments. DBHCA was identified as a chemical compound with a feature of rapid combination and dissociation, whose association (*k*_a_) and dissociation (*k*_d_) rates were 3.766 x 10^4^ (1/Ms) and 1.502 x 10^−3^ (1/s), respectively. As previously reported, an ideal medicine is supposed to bind its target with fast association and slow dissociation, which means the rapid dissociation of drug represents a safer drug selection for human or animal babesiasis, while a too fast dissociation rate could be deleterious to the medical treatment (Jiao et al., 2018). For this reason, we suggest that the compound DBHCA could be improved and optimized by suitably reducing its dissociation rate. Conversely, the DHNA compound revealed a feature of slow association and dissociation and its *k*_a_ and *k*_d_ values are 20.49 (1/Ms) and 7.718 x 10^−4^ (1/s). As slow association could delay the onset of the drug and frequently cause the deterioration of disease, an effort should be made to improve the association rate (*k*_a_) of DHNA. Therefore, the renewed structure-based development of DBHCA and NDNA might enable them to serve as new anti-*Babesia* drugs, especially the low cytotoxic compound NDNA. Available data show that the compound FX11, a derivative of gossypol, selectively inhibit the human LDH-A (*K*_i_ = 8 μM), but not the human LDH-B and glyceraldehyde-3-phosphate dehydrogenase, even at high concentrations (Deck et al., 1998; Rellinger et al., 2017). Currently, FX11 exhibits a preclinical efficacy for the treatment of cancers, including adult lymphoma cancer, pancreatic cancer and prostate cancer (Le et al., 2010).

In malaria parasites, the crystal structures of PfLDH complexed with a series naphthalene-based compounds, such as 2,6-naphthalenedicarboxylic acid, 2,6-naphthalene disulphonic acid, and 3,7-dihydroxy naphthalene-2-carboxylic acid have been resolved at high resolutions, and the complex structures revealed that the PfLDH could form the binding site for gossypol and its derivatives in two binding modes: one overlapping the substrate site but not the co-factor site, and the other bridging the binding sites for the co-factor and the substrate (Conners et al., 2005). Therefore, understanding the complex structures of BmLDH with naphthalene-based compounds could lay a structural basis for the design and development of novel babesial pharmaceuticals. To this end, we explored the complex crystal structures of BmLDH with DBHCA or DHNA in this study. Despite the success in obtaining the crystal of BmLDH apo form and solving the structure of BmLDH at a resolution of 2.79 Å (Hampton, California, USA), we failed to prepare the crystals complexed with DBHCA or DHNA using the soaking method.

It is worth noting that the naphthoic acid compounds, especially structure-based derivatives, are easily and economically accessible for artificial synthesis (Lu et al., 2017). The research on the naphthalene-based compounds or nucleophilic groups interacting with catalytic residues of the BmLDH would contribute to the discovery of new anti-*Babesia* drugs with higher efficiency and lower cost. At present, with the development of computer technology, the drug design has ushered in the era of virtual screening. Potential drugs can be predicted by computer virtual screening, molecular docking, and molecular dynamics simulation on the interactions between drug candidates and their target calculated affinities (Saxena et al., 2018; Brandao et al., 2018). Our subsequent study will focus on the virtual screening of drugs based on the pharmacophore of these compounds to discover new LDH inhibitors and specific medicines.

As the inhibitory effects of both DBHCA and DHNA are not very strong, a renewed structure-based development needs to be performed. Future study will focus on the renewed structure-based on DHNA to improve the affinity of DHNA to rBmLDH. The preliminary structural modification for the compound DHNA has been finished, and a compound library will be tested by SPR experiment to verify the modification effect. On the other hand, since both DBHCA and DHNA have the same target, they may have a synergistic interaction and the combination therapy based on both compounds could be another future scope.

## Conclusion

In conclusion, two naphthalene-based compounds DBHCA and DHNA were identified to target BmLDH and inhibit both the enzyme activity of BmLDH and the growth of *B. microti in vitro*. SPR analysis offered more novel insights into the binding properties (association and dissociation) between BmLDH and the two compounds. Additionally, cytotoxicity tests of DBHCA and DHNA in Vero cell line further demonstrated that DHNA has a higher selectivity index than DBHCA between *B. microti* and Vero cells. These findings provide some theoretical basis for renewed structure-based development of the two naphthalene-based compounds as novel anti-*Babesia* agents for treatment of human babesiosis.

## Data Availability Statement

The raw data supporting the conclusions of the article will be made available by the authors, without undue reservation, to any qualified researcher.

## Author Contributions

LY, LH, and JZ designed the study and wrote the draft of the manuscript. LY, XZ, QL, YS, ML, YZ, XA, and YT performed the experiments and analyzed the results. All authors have read and approved the final manuscript.

## Funding

This work was supported by the National Key Research and Development Program of China (2017YFD0501201), the National Natural Science Foundation of China (31772729) and the Natural Science Foundation of Hubei Province (2017CFA020).

## Conflict of Interest

The authors declare that the research was conducted in the absence of any commercial or financial relationships that could be construed as a potential conflict of interest.

## Abbreviations

BmLDH, *Babesia microti* lactate dehydrogenase; PfLDH, *Plasmodium falciparum* lactate dehydrogenase; HmLDH, human lactate dehydrogenase; PRA-99, *B. microti* ATCC PRA-99™ strain; IC_50_, half-maximal inhibitory concentration; CC_50_, half-maximal cytotoxic concentration; TCA cycle, tricarboxylic acid cycle; *k*_a,_ association constant; *k*_d_, dissociation constant; *K*_i_, inhibition constant; *K*_D,_ equilibrium dissociation constant; NDCA, 2,6-naphthalenedicarboxylic acid; DBHCA, 1,6-dibromo-2-hydroxynapthalene 3-carboxylic acid; DHNA, 3,5-dihydroxy 2-naphthoic acid; SI, selectivity index; DMSO, dimethyl sulfoxide; ORF, open reading frame; ATP, adenosine triphosphate; NADH, reduced nicotinamide adenine dinucleotide; NAD^+^, nicotinamide adenine dinucleotide; RBC, red blood cell; NHS, *N*-hydroxysuccinimide; EDC, 1-ethyl-3-(3-dimethylaminopropyl) carbodiimide hydrochloride.
